# *CARD14* mutations may predict response to antitumour necrosis factor‐α therapy in psoriasis: a potential further step towards personalized medicine

**DOI:** 10.1111/bjd.14795

**Published:** 2016-08-03

**Authors:** K.C.P. Wu, N.J. Reynolds

**Affiliations:** ^1^ Dermatological Sciences Institute of Cellular Medicine University of Newcastle Newcastle upon Tyne NE2 4HH U.K.

## Abstract

**Linked Article:** http://dx.doi.org/10.1111/bjd.14461.

There has been considerable interest in the concept of personalized or stratified medicine, in which a patient's individual characteristics, such as demographics, genotype and clinical phenotype, are used to select particular interventions or to predict response to therapy or its side‐effects.^1^ In some cases personalized medicine is already a reality and used in everyday practice. Dermatologists are currently recommended to assay for thiopurine methyltransferase (TPMT) activity prior to optimizing azathioprine treatment, in order to avoid it in those who are null for TPMT.^2^ Furthermore, in patients with late‐stage melanoma, dermato‐oncologists screen for the BRAF V600E mutation prior to prescribing vemurafenib.

Advances in pharmacogenetic studies have demonstrated that particular genetic variations may be associated with clinical response or hypersensitivity to therapy. For example, there is a strong association between the human leucocyte antigen (HLA)‐B*5701 allele and hypersensitivity to abacavir, a reverse‐transcriptase inhibitor used to treat human immunodeficiency virus infection: odds ratio 117; 95% confidence interval 29–481.^3^ The HLA‐B*5701 allele has a prevalence of 5–7% in Western Europe, and screening for this allele has previously been shown to reduce the incidence of adverse reactions to abacavir.^4^ The U.S. Food and Drug Administration now recommends HLA‐B*5701 testing when considering abacavir therapy; patients who test positive should be offered an alternative treatment.

Although genetic studies have increased the number of known psoriasis susceptibility loci in individuals of European ancestry to 41,^5^ factors that predict response to psoriasis therapy have only recently begun to emerge. For example, in 2013 Talamonti *et al*. reported that the HLA‐Cw*0602 allele, but not the LCE3C_LCE3B deletion, is associated with a clinical response to ustekinumab, an anti‐interleukin‐12/23 monoclonal antibody.^6^

In this issue of the *BJD*, Coto‐Segura *et al*. link polymorphisms in the caspase recruitment domain family member 14 gene (*CARD14*), which activates nuclear factor (NF)‐κB, to clinical response to tumour necrosis factor (TNF)‐α‐blocking agents including adalimumab, etanercept and infliximab.^7^ Their study of 116 patients with psoriasis showed that the common *CARD14* rs11652075 polymorphism (Arg820Trp) was associated with a positive response to anti‐TNF‐α therapy over 24 weeks: odds ratio 3·67, 95% confidence interval 1·37–9·84.^7^ Interestingly the rs11652075 polymorphism in *CARD14* has been associated with increased risk of psoriasis,^8^ and Coto‐Segura *et al*. suggest that polymorphisms in *CARD14* may lead to enhanced NF‐κB/TNF‐α pathways and thus increased responsiveness to anti‐TNF‐α treatment.^7^ However, the functional consequences of the missense single‐nucleotide polymorphism rs11652075 (Arg820Trp) in *CARD14* require further characterization.

Although replication of these results in a prospective large cohort would be important, this study provides some further support that pharmacogenetics may one day be able to predict clinical response (and potentially hypersensitivity) to psoriasis treatment. Ultimately this could lead to more efficient use of healthcare funding and decreased incidence of (potentially life‐threatening) side‐effects.

## Conflicts of interest

K.C.P.W. undertook a Wellcome Trust‐funded PhD, with GlaxoSmithKline (GSK) acting as an industrial partner, although he did not receive salary support from GSK. N.J.R. has received consulting fees, consulting income (Newcastle University), research grants (Newcastle University) and travel support from AbbVie, Amgen, Celgene, Novartis, Pfizer and Stiefel GSK. Neither of the authors has received direct support from any organization for the submitted work. The research of N.J.R. is supported by the National Institute for Health Research through Newcastle's Biomedical Research Centre.
